# Supplementary material for: A human H5N1 influenza virus expressing bioluminescence for evaluating viral infection and identifying therapeutic interventions
Source: iScience. 2025 Aug 21;28(9):113402. doi: 10.1016/j.isci.2025.113402 (PMC12446200; doi:10.1016/j.isci.2025.113402)
Supplement: Document S1. Figure S1, Table S1, and Data S1 [file mmc1.pdf]

iScience, Volume *28*

Supplemental information

A human H5N1 influenza virus expressing bioluminescence for evaluating viral infection and identifying therapeutic interventions

Ramya S. Barre, Ruby A. Escobedo, Esteban M. Castro, Michal Gazi, Joshua D. Castro, Anastasija Cupic, Mahmoud Bayoumi, Nathaniel Jackson, Chengin Ye, Aitor Nogales, Roy N. Platt, Ricardo Carrion Jr., Timothy J.C. Anderson, Adolfo García-Sastre, Ahmed Mostafa, and Luis Martinez-Sobrido

Gene		Coverage and SNP(S)
PB2	Coverage	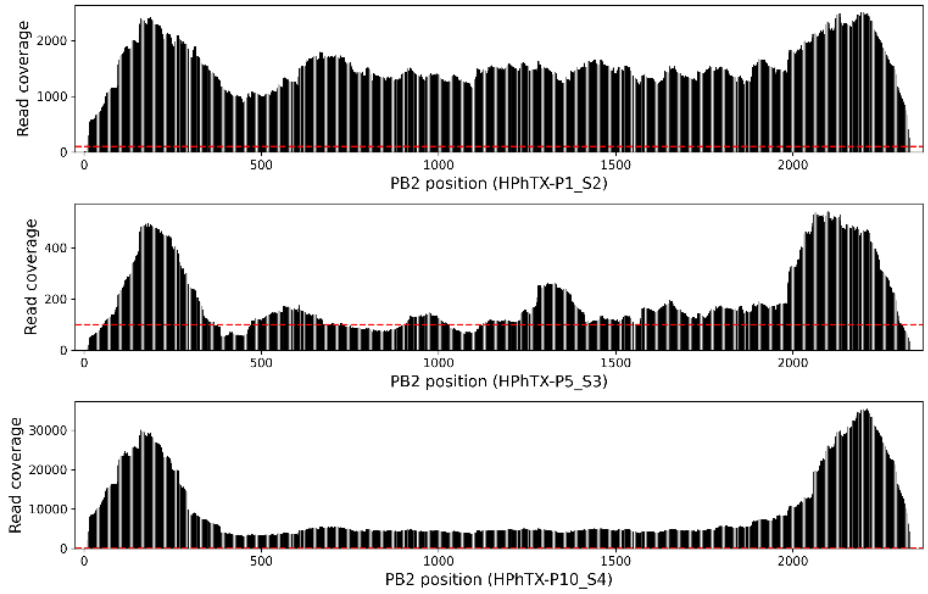
	SNP(S)	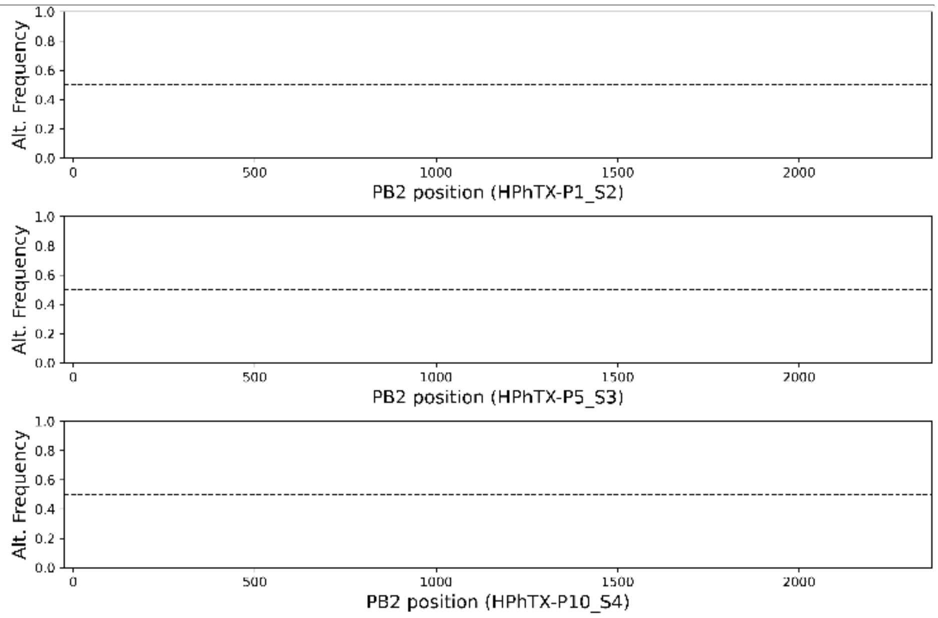

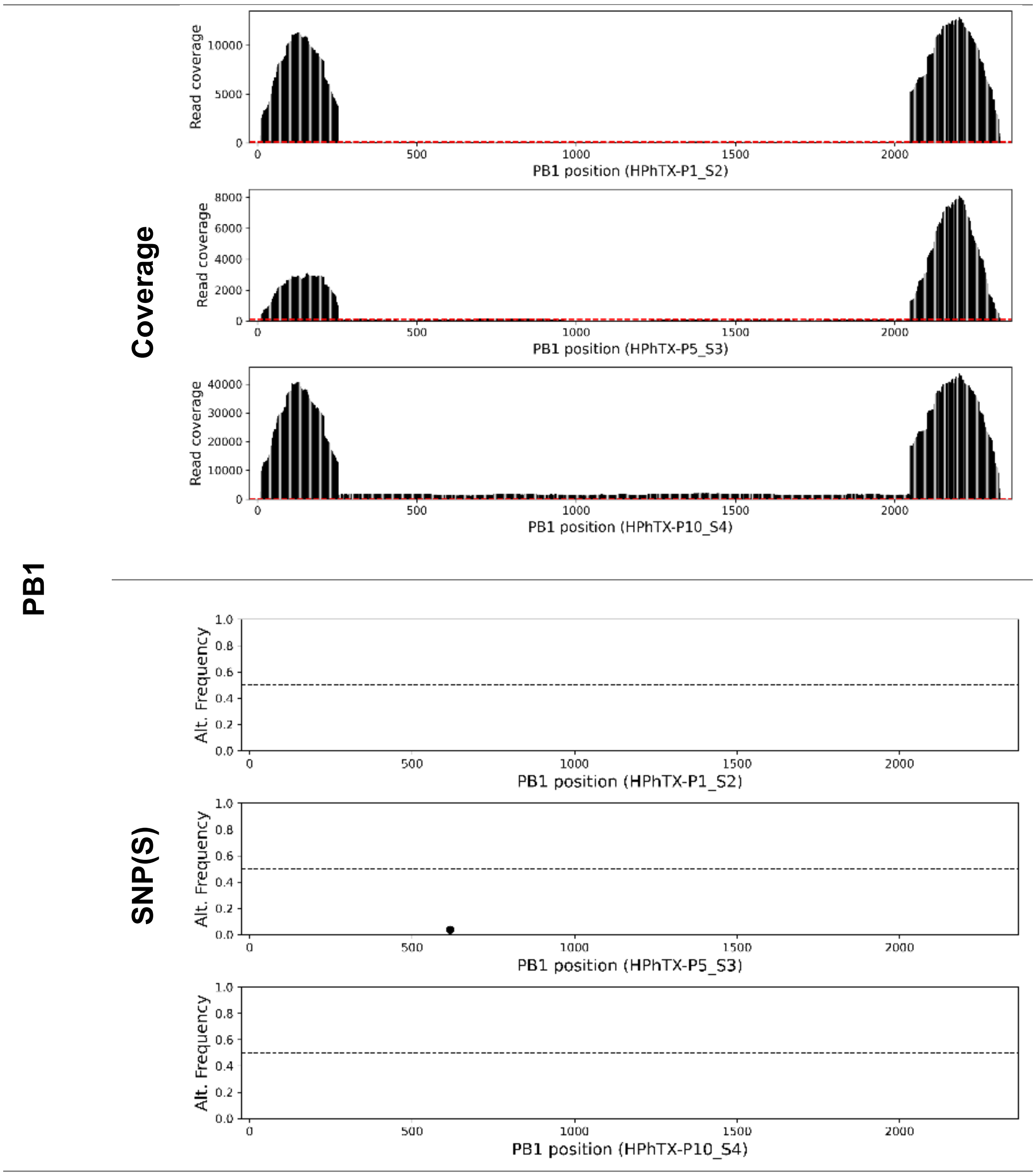
PB1
Coverage
Read coverage
PB1 position (HPhTX-P1_S2)
PB1 position (HPhTX-P5_S3)
PB1 position (HPhTX-P10_S4)
SNP(S)
Alt. Frequency
PB1 position (HPhTX-P1_S2)
PB1 position (HPhTX-P5_S3)
PB1 position (HPhTX-P10_S4)

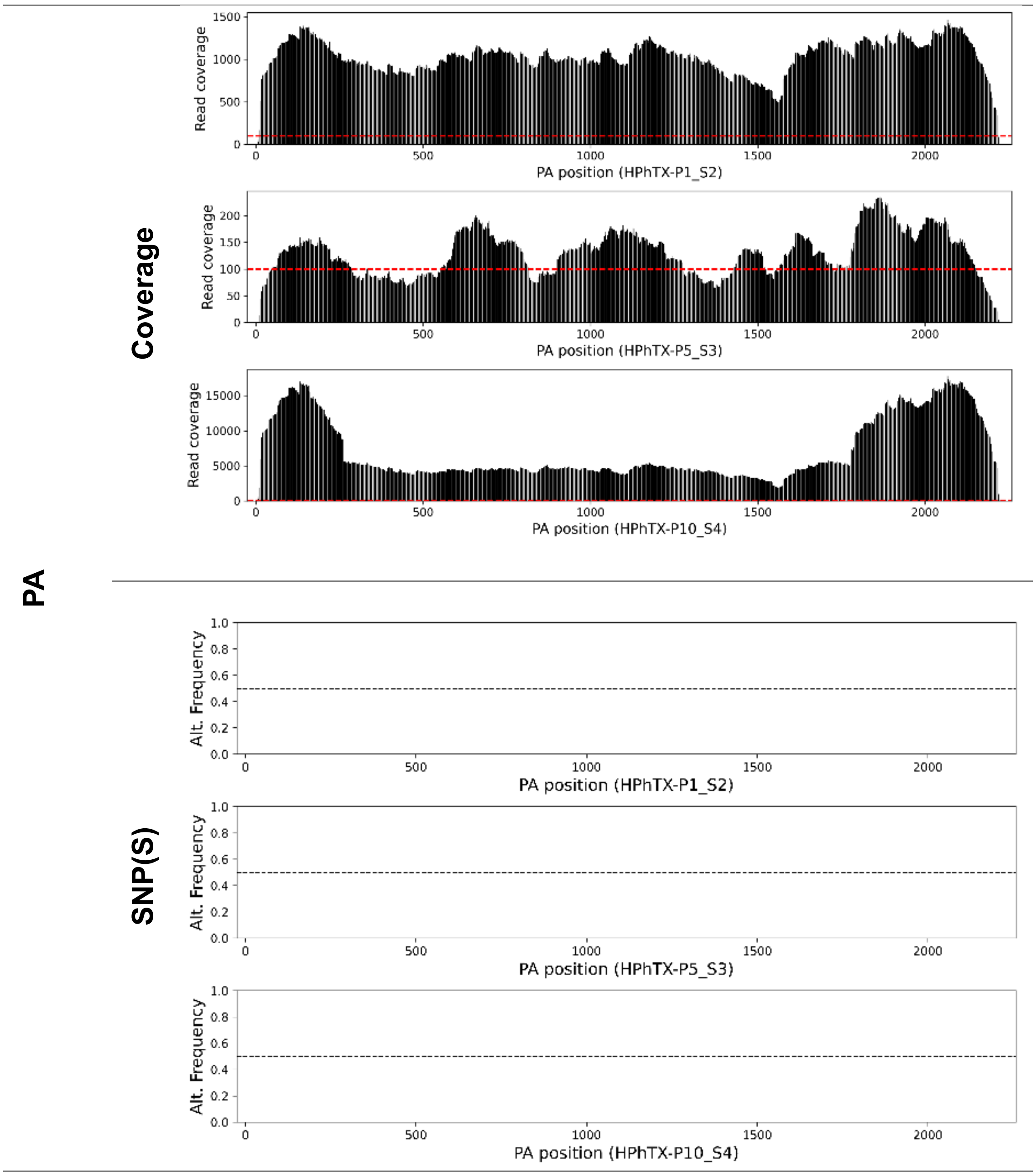
PA
Coverage
SNP(S)
Read coverage
PA position (HPhTX-P1_S2)
PA position (HPhTX-P5_S3)
PA position (HPhTX-P10_S4)
Alt. Frequency
PA position (HPhTX-P1_S2)
PA position (HPhTX-P5_S3)
PA position (HPhTX-P10_S4)

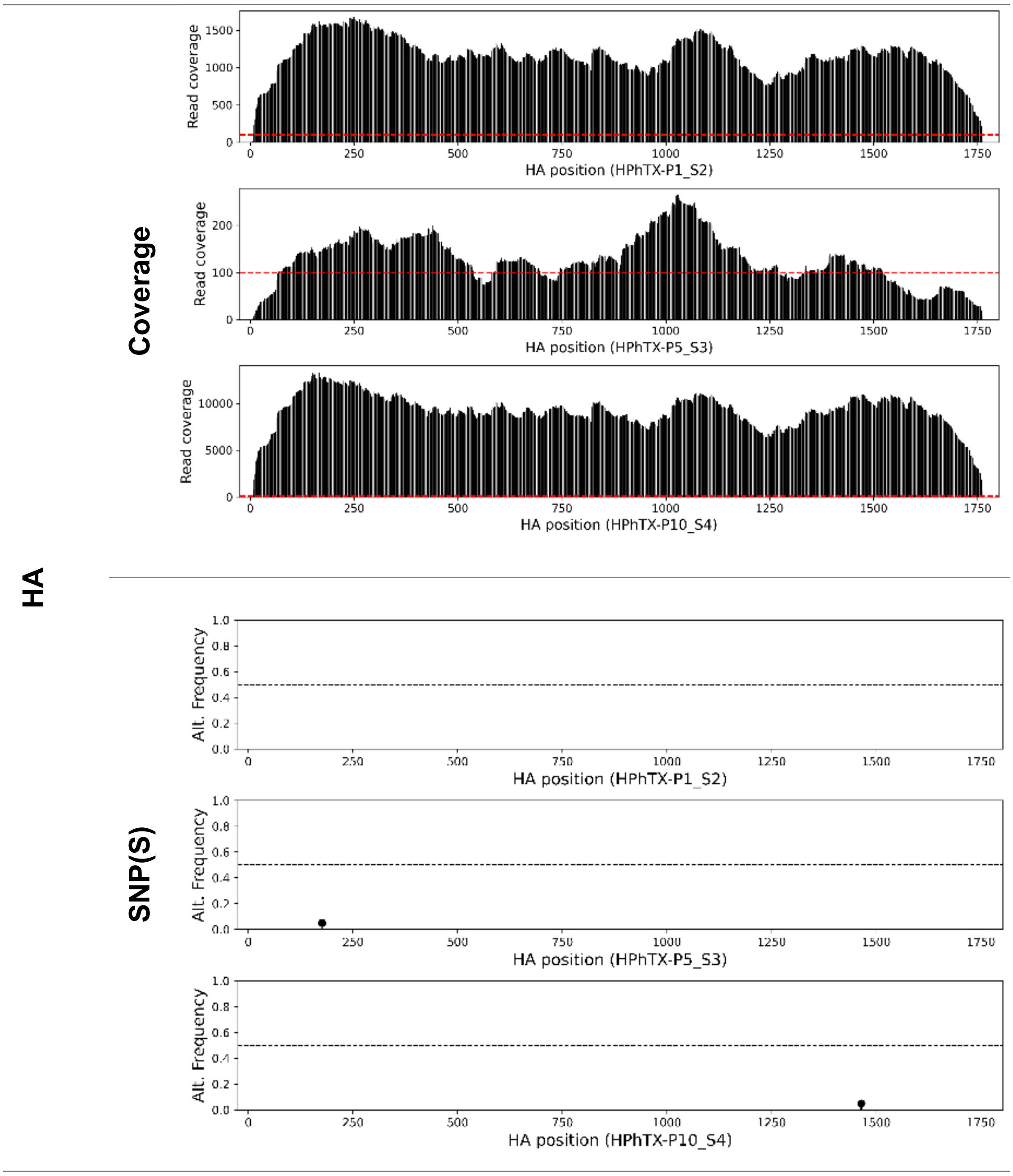

HA
Coverage
Read coverage
HA position (HPhTX-P1_S2)
HA position (HPhTX-P5_S3)
HA position (HPhTX-P10_S4)
SNP(S)
Alt. Frequency
HA position (HPhTX-P1_S2)
HA position (HPhTX-P5_S3)
HA position (HPhTX-P10_S4)

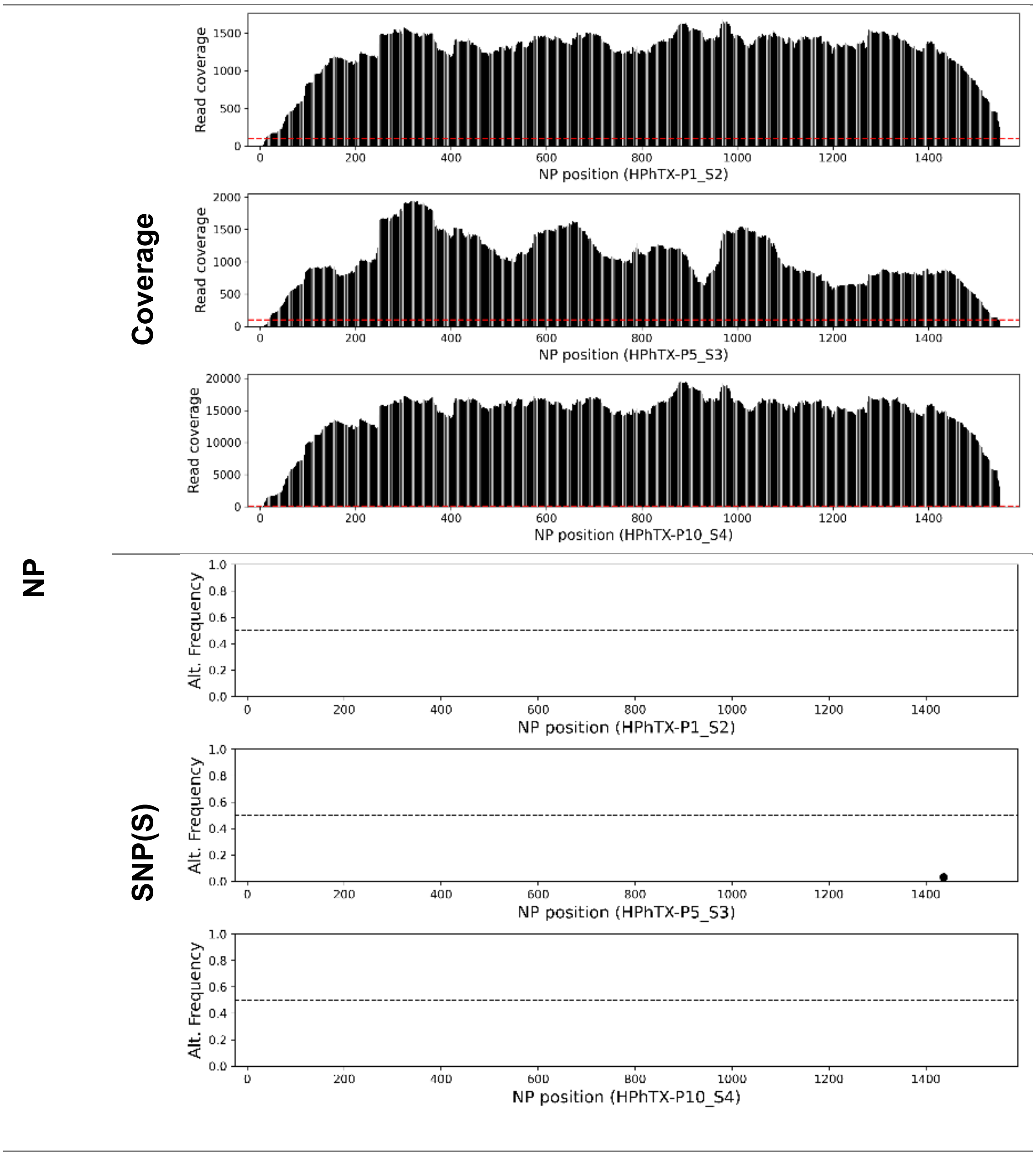
NP
Coverage
SNP(S)
Read coverage
NP position (HPhTX-P1_S2)
NP position (HPhTX-P5_S3)
NP position (HPhTX-P10_S4)
Alt. Frequency

NA

Coverage

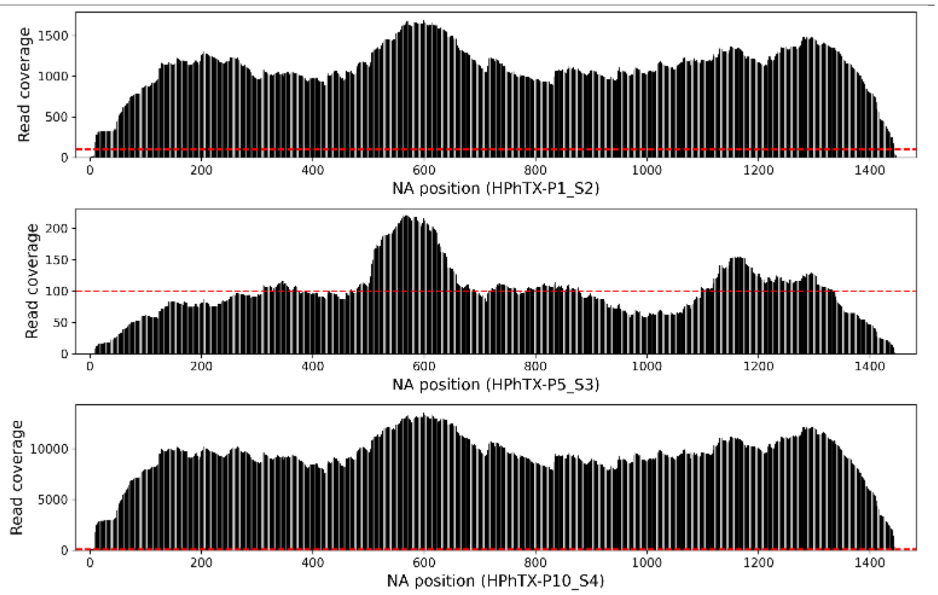

SNP(S)

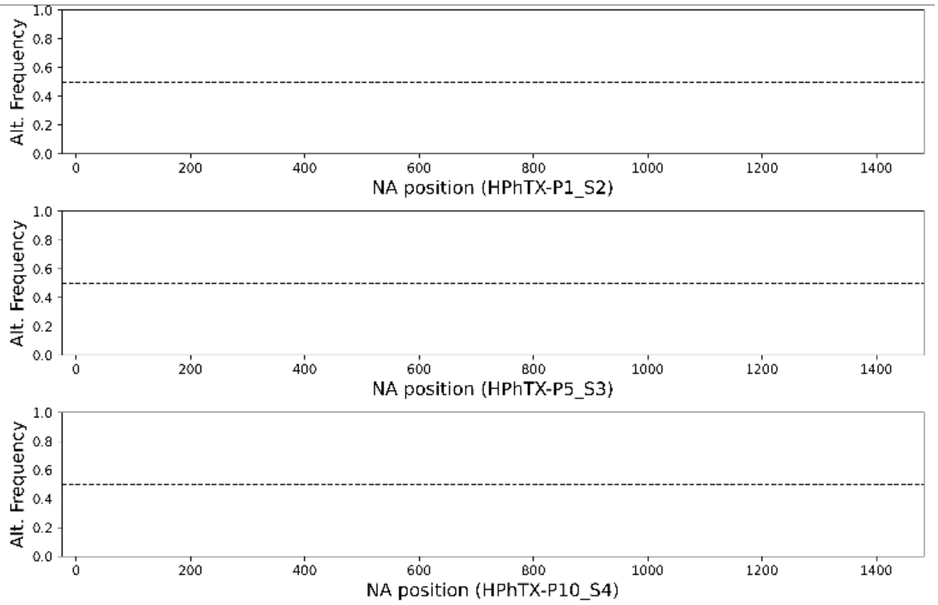

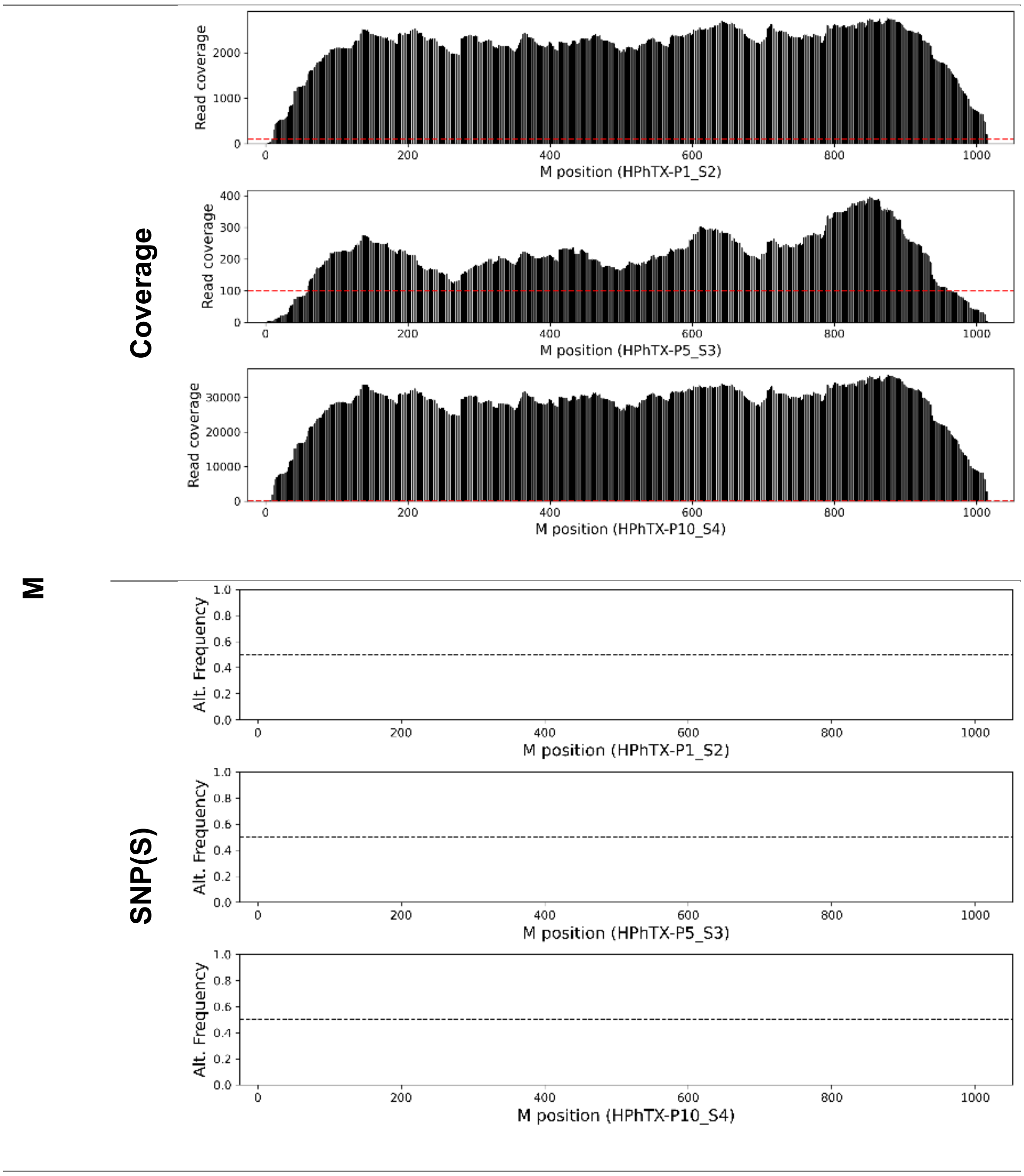

Data S1. Genetic coverage and single nucleotide polymorphisms (SNPs) of HPhTX NSs-Nluc viral segments PB2, PB1, PA, HA, NP, NA, and M following *in vitro* passaging in MDCK cells.

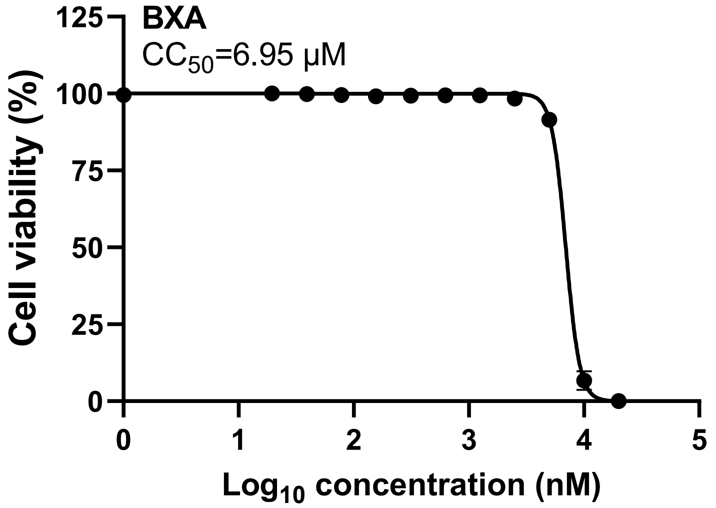

Figure S1. Baloxavir (BXA) cytotoxicity in MDCK cells. MDCK cells were incubated with 10-fold serial dilutions (starting concentration 10 µM) of BXA. At 48h, cell monolayers were fixed with 4% paraformaldehyde (PFA) solution for 20 min and then stained with 0.1% crystal violet staining solution. The dry crystal violet stains were dissolved in 100% methanol and read at 570 nm. Error bars represent standard error of the mean (SEM), with each experiment undertaken independently in quadruplicate. The 50% cytotoxic concentration (CC_{50}) of BXA was obtained by the crystal violet assay and calculated using nonlinear regression analysis of GraphPad Prism software (version 5.01) by plotting log inhibitor versus normalized response (variable slope).

Table S1. Variant frequencies - All variants at <25% allele frequency are shown.

**Sample**	**Segment**	**Pos**	**Ref. allele (amino acid)**	**Alt. allele (amino acid)**	**Alt. freq**	**Read Depth**
**HPhTX-P10**	HA	712	A (Lysine)	C (Asparagine)	5.78%	9969
**HPhTX-P10**	HA	1464	G (Glycine)	A (Aspartic acid)	4.87%	9667
**HPhTX-P5**	PA	753	G (Glutamic acid)	A (Glutamic acid)	5.35%	187
**HPhTX-P10**	PA	1078	G (Glutamic acid)	A (Lysine)	3.04%	5597
**HPhTX-P10**	PA	1093	A (Threonine)	C (Proline)	13.50%	5349
**HPhTX-P5**	PB1	617	A (Lysine)	G (Arginine)	3.73%	161
**HPhTX-P10**	PB1	1227	C (Alanine)	A (Alanine)	3.20%	2188
**HPhTX-P10**	PB2	181	G (Alanine)	A (Threonine)	11.73%	9911
**HPhTX-P10**	PB2	267	G (Lysine)	A (Lysine)	33.23%	9961
